# Sentential Context Modulates the Involvement of the Motor Cortex in Action Language Processing: An fMRI Study

**DOI:** 10.3389/fnhum.2013.00100

**Published:** 2013-04-08

**Authors:** Karen D. I. Schuil, Marion Smits, Rolf A. Zwaan

**Affiliations:** ^1^Erasmus University RotterdamRotterdam, Netherlands; ^2^Department of Radiology, Erasmus MC – University Medical Center RotterdamRotterdam, Netherlands

**Keywords:** action simulation, embodied cognition, fMRI, semantic somatotopy, language comprehension

## Abstract

Theories of embodied cognition propose that language comprehension is based on perceptual and motor processes. More specifically, it is hypothesized that neurons processing verbs describing bodily actions, and those that process the corresponding physical actions, fire simultaneously during action verb learning. Thus the concept and motor activation become strongly linked. According to this view, the language-induced activation of the neural substrates for action is automatic. By contrast, a weak view of embodied cognition proposes that activation of these motor regions is modulated by context. In recent studies it was found that action verbs in literal sentences activate the motor system, while mixed results were observed for action verbs in non-literal sentences. Thus, whether the recruitment of motor regions is automatic or context dependent remains a question. We investigated functional magnetic resonance imaging activation in response to non-literal and literal sentences including arm and leg related actions. The sentence structure was such that the action verb was the last word in the subordinate clause. Thus, the constraining context was presented well before the verb. Region of interest analyses showed that action verbs in literal context engage the motor regions to a greater extent than non-literal action verbs. There was no evidence for a semantic somatotopic organization of the motor cortex. Taken together, these results indicate that during comprehension, the degree to which motor regions are recruited is context dependent, supporting the weak view of embodied cognition.

## Introduction

There are two major views regarding the organization of conceptual representations in the human brain. The traditional view in cognitive science treats concepts as abstract, symbolic, amodal entities (e.g., Fodor, 1975; Kintsch and Van Dijk, 1978). It emphasizes that concepts are represented independently of the brain’s sensorimotor system. More recent theories have emphasized an important role for sensorimotor information in the organization of conceptual knowledge (e.g., Barsalou, 1999). This view is known as “embodied cognition” and has for example been supported by behavioral and neuroimaging findings indicating that understanding action language engages action planning systems. Numerous behavioral studies have shown interactive effects between language comprehension and action execution. For example, Glenberg and Kaschak (2002) showed that hand movements toward or away from the body were facilitated by sentences describing a congruent action (e.g., “He opened/closed the drawer,” respectively), compared to when the arm movement was incongruent with the sentence. Similarly, Zwaan and Taylor (2006) found that sentences describing manual rotation (e.g., He turned down/up the volume) facilitated the manual rotation of a knob (to the left/right, respectively). In turn, manual rotation of the knob facilitated the reading of sentences that implied a congruent rotation.

Neuroimaging studies have provided converging evidence of how action verbs in literal sentences are processed. Processing action verbs and action sentences recruits the premotor cortex [PM, i.e., Brodmann area (BA) 6] in a manner similar to the direct observation or execution of actions. For instance, Hauk et al. (2004) found that primary motor cortex (M1, i.e., BA 4), and PM activation associated with reading action words related to leg (kick), arm (pick), and face (lick) actions and execution of foot, fingers, and tongue movements partially overlapped. Interestingly, leg and arm related action verbs activated M1 and hand and face related verbs activated PM in a somatotopic fashion. In other words, the activation spatially differed within the M1 and PM according to their known somatotopic organization, depending on whether the words denoted a hand, face, or leg action. For example, reading about hand related actions (e.g., *to throw*) activates the hand area more so than the foot area. The latter area is more activated by reading about foot related actions than reading about hand related actions.

These studies demonstrated that reading action verbs engages the motor cortex. The question here is whether when action verbs are placed in a context they are associated with similar activation of the motor cortex. Action verbs like *greifen* (to grasp) compared to abstract verbs activated the sensorimotor cortex and secondary somatosensory cortex (Rueschemeyer et al., 2007). However, when comparing morphologically complex verbs like *begreifen* (to comprehend) with abstract verbs no such activation was found. Even though these complex action verbs have a motor stem they were processed as non-literal language. In addition, Aziz-Zadeh et al. (2006) investigated similarities and differences between action observation and reading of hand, leg, and mouth actions in a sentential context. Processing literal action sentences, like *Grasping the scissors* and observing the same action was related to activation in the PM. Furthermore, they found overlapping effector-specific activations for action observation and action reading. Other studies have also found that semantic processing of action verbs related to different body parts evokes somatotopically specific activation in motor regions (e.g., Willems et al., 2009). More specifically, when left and right handers performed a lexical-decision task to manual action verbs (compared to non-manual action verbs) they activated the right PM and left PM, respectively. It is often discussed whether activation in PM associated with action language is the result of motor imagery. Recently, Willems et al. (2010) found that PM activation was related to action simulation and not to imagery. They found that during a lexical-decision task hand related action verbs (compared to non-manual actions) activated PM and not M1. Imagery on the other hand activated both PM and M1. In light of these findings, the M1 and PM activation found by Hauk et al. (2004) could suggest that the verbs without context are processed as an order. Language simulation seems to activate only the PM, whereas imagery also activates M1 (Willems et al., 2010). Furthermore, during imagery a body specific activation was found in M1. This was not found for the lexical-decision task, i.e., during simulation. Likewise, other studies using functional magnetic resonance imaging (fMRI; Tomasino et al., 2007) found enhanced M1 activation during explicit imagery of short motor related phrases (compared to non-motor phrases) compared to a letter detection task of motor related phrases (compared to non-motor phrases), and transcranial magnetic stimulation (TMS; Tomasino et al., 2008) found activation of M1 during explicit mental motor imagery but not in a frequency judgment or silent reading of hand related action verbs. These findings of motor activation in response to reading action verbs are extended to reading action sentences. Tettamanti et al. (2005) for example found motor activation in response to listening to sentences with literal mouth, hand, or leg related actions.

The theoretical framework of embodied cognition can be broadly divided into two versions (Chatterjee, 2010). In the strong version all concepts, even seemingly abstract ones (e.g., argument is war), are grounded in and interrelated with sensorimotor experience. According to this view, even when an action verb occurs in a non-literal context, the understanding of it should recruit the motor areas. This view predicts for example that reading *he kicked the habit* involves motor activation (Lakoff and Johnson, 1980; Gallese and Lakoff, 2005). In contrast, a weak embodiment view assumes that the motor system is recruited only when concepts are related to physical actions (e.g., *kicking a ball*). In the instance of a non-literal sentence with a subject-object-verb order (e.g., *the habit kicked*), the verb will be processed more by the abstract system than the sensorimotor system, unless the sentence is perceived as literal (e.g., by non-native speakers of Dutch). According to the weak embodiment hypothesis motor activation is necessary for optimal comprehension of action language (Taylor and Zwaan, 2009). Several studies have found that this activation appears to contribute to comprehension (see Taylor and Zwaan, 2009 and for a review; Casteel, 2011). In other words, the weak account does not exclude the existence of an abstract system, but argues that sensorimotor activation is necessary for optimal comprehension of action language. Imaging studies regarding non-literal action language have yielded inconsistent results. Many studies have demonstrated activation in the PM for literal action sentences, but not for idiomatic ones (Aziz-Zadeh et al., 2006; Raposo et al., 2009; Desai et al., 2010, 2011). Boulenger et al. (2009), on the other hand, found activation in PM and M1for both non-literal and literal action sentences involving leg and arm verbs.

In the present study, we asked whether motor regions are automatically involved in the processing of action words or whether the activation of the sensorimotor cortex is context dependent. We investigated fMRI activation in response to non-literal and literal sentences including arm and leg related actions. To ensure that the context was fully processed before the action verb appeared, we always presented the verb at the end of the sentence. Furthermore, we examined whether action verbs activated the M1 and/or PM in a somatotopic fashion.

One potential source of the mixed results that exist in the literature concerning motor activation related to non-literal sentences is the location of the action verb relative to the context. In several studies the context was presented after the verb (Aziz-Zadeh et al., 2006; Boulenger et al., 2009). Consider the sentence *He kicked the habit*. Only at the last word of the sentence we do learn that the verb does not denote a motor act. It is conceivable that the non-literal context did not have sufficient time to constrain verb-based motor activation. Therefore, it is necessary to provide a stronger test to detect the presence of motor activation in non-literal contexts. The Dutch language is highly suitable for this purpose. Although Dutch is a subject-verb-object (SVO) language in main clauses, it uses an SOV order in subordinate clauses. An example is *Iedereen was blij toen oma een ander onderwerp aansneed*, which literally translates to *Everyone was happy when grandma another topic cut*. In this sentence, the context is presented before the action verb, *cut* (meaning *broached* in the context of the sentence), which appears at the very end of the sentence. Only at this point is it clear that the context is non-literal. Therefore, such sentences provide the strongest possible test for the strong-embodiment claim that motor activation occurs even in non-literal contexts.

A second important aspect of this study is that we investigated semantic somatotopy using regions of interest (ROIs) that were both cytoarchitectonically (i.e., structurally) and functionally defined. Structural definition was done to ensure that we used ROIs in the regions (BA 4 and BA 6) where previous neuroimaging studies on action execution and action observation have demonstrated somatotopy (e.g., Buccino et al., 2001), enabling a comparison of our results to those previously reported. However, across studies on action words or action sentences the reported peaks of activation were usually not within the cytoarchitectonic boundaries of the motor areas (see Postle et al., 2008). In addition there is little overlap of effector-specific peak activation. We therefore combined the structurally and functionally defined ROIs, to account for such variability that may – in part – be due to incongruence of structural and functional anatomy. The functional ROIs were based on a motor localizer task, involving hand and foot movement.

In sum, the current study has several advantages over other studies. One advantage is that the verb is always the last word of the sentence. Thus the context is presented before the verb. Second, ROIs are both structurally and functionally defined.

The specific details of the present study, when viewed in the context of the two theories of embodied cognition mentioned before, lead to two hypotheses for each theory. The strong-embodiment hypothesis predicts that all action-related concepts activate the motor system equally, and does not distinguish between abstract and concrete concepts. Hence, according to this theory, PM is automatically activated when reading about actions, irrespective of level of comprehension. With regard to the semantic somatotopy prediction spatially effector-specific activation is expected. In other words, a main effect of extremity (hand/foot) related action verb is expected, meaning that hand related action verbs are expected to elicit more motor activation than foot related action verbs within the hand area and that foot related action verbs will elicit more motor activation than hand related action verbs within the foot area. Secondly, according to the weak embodiment hypothesis context is important. According to this theory, PM is only activated by an action verb in a literal and not a non-literal context. With regard to effector-specific activation a main effect of extremity related action verb and an interaction between extremity and sentence type (non-literal/literal) are expected.

Furthermore, the finding that M1 activation occurs during mental imagery and not during mental simulation (Willems et al., 2010), and because the current task reflects simulation rather than imagery, we expect to find stronger motor activation in PM (BA 6) than in M1 (BA 4). Because the verb is presented at the end of the sentence (after the context), this study provides a strong test for whether motor activation occurs in response to action verbs in non-literal context.

## Materials and Methods

### Subjects

We tested 20 healthy, native Dutch-speaking undergraduate students of the Erasmus University Rotterdam (10 male; mean age = 22.1 years; range = 18–25 years) without neurological impairments, dyslexia, or other language-related problems or hearing complaints and with normal or corrected-to-normal vision. Furthermore, participants had gone to school in the Netherlands and reported that they spoke Dutch at home; hence subjects could be expected to have good comprehension of non-literal language. All participants were right-handed, as measured by the Dutch version of the Edinburgh Handedness Inventory (EHI; Oldfield, 1971; Van Strien, 1992; *M* = 9.65, range 6–10) and gave written informed consent prior to scanning. Two participants were excluded from the ROI analysis, because their localizer data were lost due to a server crash. The study was approved by the Ethics Committee of the Erasmus MC – University Medical Center Rotterdam.

### Materials

Stimuli consisted of 200 Dutch sentences (non-literal/literal and foot/hand-related: “non-literal foot”, “non-literal hand”, “literal foot”, and “literal hand”) and 50 unpronounceable non-word sentences (baseline condition), resulting in 50 sentences per condition (see example sentences in Table 1). The sentence structure was such, that the context was clear before the verb appeared. Hand and foot action sentences were used to investigate semantic somatotopy. An analysis of variance (ANOVA) showed that the mean number of words, syllables, and characters of the four conditions (“non-literal foot”, “non-literal hand”, “literal foot”, “literal hand”) did not differ significantly across conditions (*p*s > 0.05; see Table 2).

**Table 1:** **Example sentences and their literal translation**.

Sentence type	Example	Mean number of characters/sentence (SD)	Syllables	words
Non-literal foot	De student had het tentamen toch gehaald, ondanks dat hij op zijn tenen liep	57.80 (8.49)	18.64 (2.82)	12.96 (1.65)
	The student had passed the exam, even though he on his toes walked	
Non-literal hand	Lia was een pechvogel die altijd aan het kortste eind trok	57.44 (8.26)	18.5 (2.90)	12.42 (1.94)
	Lia was an unlucky person, who always the shortest end pulled	
Literal foot	De havenwerker zag dat zijn collega een beetje mank liep	57.42 (7.47)	18.42 (2.79)	12.52 (1.47)
	The dock laborer saw that his colleague a bit crippled walked	
Literal hand	Frans was zo verstandig dat hij een regenpak aantrok	56.92 (9.01)	18.34 (2.95)	12.58 (2.06)
	Frans was so wisely that he a rain suit tighter pulled	
Baseline	Pg umoyod tppd sf pcsox wpm rdrq djg agih ht eahme swrdsbmvgq			
	No translation available	

**Table 2:** **Means for each sentence type**.

Sentence type	Mean number of words (SD)	Mean number of syllables (SD)	Mean number of characters/sentence (SD)
Non-literal hand	12.96 (1.65)	18.64 (2.82)	70.10 (15.62)
Non-literal foot	12.42 (1.94)	18.50 (2.90)	69.22 (15.59)
Literal foot	12.52 (1.47)	18.42 (2.79)	69.42 (14.29)
Literal hand	12.58 (2.06)	18.34 (2.95)	68.74 (15.37)

### Procedure

Stimuli were presented visually using Presentation software (Neurobehavioral Systems, Albany, CA, USA version 14.6) through a projector from outside the scanner room by rear-projection onto a screen at the front of the scanner bore and were visible to the participants through a mirror attached to the head coil. Participants were instructed to read the sentences silently. To make sentence presentation more natural the sentences were presented via a Variable Serial Visual Presentation (VSVP) procedure (Otten and Van Berkum, 2008). The duration for the word presentation in milliseconds was 187 + 27 × number of characters, with a maximum presentation time of 450 ms. The inter word interval was 106 ms and the inter trial interval was 2000 ms. The verb was presented with a fixed duration of 600 ms. The order of the sentences was pseudo-randomized and presented in two fMRI runs, making certain that each condition was not presented more than three times in a row. To ensure attentiveness during reading subjects had to press a button for on average every two and a half sentences to indicate whether a consecutively presented word described the sentence or not. In other words, participants read sentences and for approximately every second or third sentence (baseline, literal, and non-literal) a probe word was presented 2 s after the sentence. Participants pressed a button to indicate that the word was related to the meaning of the sentence and pressed another button to indicate that the word was not related to the meaning of the sentence. For the baseline sentences a letter string from the sentence appeared as a probe word. By requiring the participants to respond equally often to all sentences (including the baseline condition) we ensured that the motor responses to the task would not contaminate the results of interest. In addition, the button responses were required only for half to a third of all sentences. Thus, participants did not know when they had to respond and therefore the motor activation is unlikely to stem from attentional demands or motor preparation.

A structural scan was acquired in between the two functional runs. At the end of the session, subjects engaged in an action execution localizer task in which they performed hand movements (opening and closing the hand) and foot movements (flexing and bending the ankles and toes). The localizer task was a blocked design consisting of 20 s blocks of each of the four conditions (left hand, right hand, left foot, and right foot movement) repeated four times in pseudo-random order. Compliance with the task was visually checked from the scanner control room.

After scanning, participants filled out a non-literal sentence comprehension questionnaire for the 100 non-literal expressions used in this experiment. The questionnaire was a paper and pencil test. Participants read each non-literal expression and wrote down the meaning of that expression.

### Behavioral data

Accuracy (mean correct responses) and reaction times (correct responses) to the probe word were calculated. Performance across conditions was compared using repeated measures analysis of variance (RMA). Second responses (button presses) were excluded from the analysis. Only the first responses were judged as correct or incorrect and were fed into an RMA. Difference scores between the end and beginning of a run were calculated and a paired *t*-test was conducted to test for differences in performance presumably due to fatigue.

#### Non-literal sentence comprehension questionnaire analysis

Four independent raters judged the correctness of the participants’ descriptions of the meaning of non-literal. If the gist of the description matched the meaning of the non-literal expression an item was judged as correct. An interrater reliability analysis, using the κ statistic, was performed to determine consistency among raters.

### MRI data acquisition and analysis

Blood oxygen level-dependent (BOLD) fMRI data were acquired on a 3-T General Electric Healthcare (HDx platform, Milwaukee, WI, USA) scanner. Functional T2*-weighted images were acquired in 34 axial slices (thickness = 3.50 mm, no gap, repetition time (TR) = 2 s; field of view (FOV) = 22 cm; voxel size = 3.40 mm × 3.40 mm × 3.50 mm; matrix size = 64 × 64). To minimize effects of scanner signal stabilization the first five images were omitted from all analyses. In each run 545 volumes were acquired. For the anatomical reference scan, a 3D high-resolution inversion recovery fast spoiled gradient recalled echo T1-weighted sequence was used (192 slices, effective slice thickness = 0.80 mm, FOV = 250 mm, voxel size = 0.50 mm × 0.50 mm in-plane resolution). A high pass filter (cutoff period 128 s) was incorporated into the model to remove noise associated with low frequency confounds. Foam pads were used to restrict head movement.

The data analyses were done using SPM 8 (Wellcome Institute of Cognitive Neurology, London, UK), implemented in Matlab version 7.10 (Mathworks Inc, Sherborn, MA, USA). Preprocessing involved realignment through rigid body registration to correct for head motion, coregistration of the anatomical scan to the mean T2*-weighted image, segmentation, normalization to Montreal Neurological Institute (MNI) space (interpolation of voxel size to 2 mm × 2 mm × 2 mm), and spatial smoothing with a three dimensional full-width-half-maximal Gaussian kernel of 8 mm. Structural scans were normalized to MNI space with an interpolation of voxel size to 1 mm × 1 mm × 1 mm. Conditions (see below) for each subject were modeled with the general linear model (GLM) and convolved with the canonical hemodynamic response function. In all analyses responses (button presses) were modeled as a regressor of no interest. Two whole brain analyses (whole sentence and action verbs) and two ROI analyses (structurally and subject-specific) were performed. All clusters that passed the family wise error (FWE) correction for multiple comparisons at *p* < 0.05 are reported.

#### Localizer task

Stimuli in the action execution localizer task were modeled as blocks of 20 s (see Hauk et al., 2004). The four conditions (“left hand”, “left foot”, “right hand”, and “right foot”) were modeled as events. These contrasts were calculated at the single subject level and were fed into a second level RMA with subject as random factor. We used the contrasts “right hand” > “left hand” and “right foot” > “left foot” to determine the peak activation within BA 4 (i.e., primary motor cortex, BA4a combined with BA4p; Geyer et al., 1996) and BA 6 (i.e., PM, Eickhoff et al., 2005). These cytoarchitectonic maps were derived from the SPM anatomy toolbox (Eickhoff et al., 2005). These two contrasts were used to identify the peak activation in the left hemisphere, because predominantly left hemispheric language processing was expected in our right-handed subjects.

#### Whole brain analysis sentences

The onset and the duration of the sentences in the conditions “non-literal foot”, “non-literal hand”, “literal foot”, “literal hand”, and “baseline” were modeled. These conditions were calculated at the single subject level and were fed into a second-level whole-brain group analysis; RMA with sentence type as factor (literal, non-literal, and baseline) and subject as random factor was carried out. With this analysis we tested whether reading of non-literal as well as literal sentences activated a common cortical language network, by looking at the contrast: “all sentences” > “baseline.”

#### Whole brain analysis verbs

Every condition, “non-literal foot”, “non-literal hand”, “literal foot”, “literal hand”, “baseline” (last non-word of the sentence), was modeled as two separate events: the onset and duration of the verb and the onset and duration of the sentence up to the verb as a regressor of no interest. A second level whole brain group analysis (RMA) with sentence type as factor (“non-literal foot”, “non-literal hand”, “literal foot”, “literal hand”, and “baseline”) was carried out. With this analysis we tested whether reading of action verbs in literal sentences engaged PM to a greater extent than action verbs in non-literal sentences, by looking at the contrast of the verbs “literal” > “baseline”, “non-literal” > “baseline”, “literal” > “non-literal”, and “non-literal” > “literal”, “literal hand” > “non-literal hand”, “literal foot” > “non-literal foot”, “non-literal hand” > “literal hand”, and “non-literal foot” > “literal foot”, “foot” > “hand”, and “hand” > “foot”. In addition, we checked whether reading of the verbs in non-literal and literal contexts activated a language network, by looking at the contrast “all verbs” > “baseline.”

#### ROI analyses

The type of errors on the sentence questionnaire did not indicate that participants comprehended any of the sentences as literal, and therefore only the verbs of sentences that participants did not know the meaning of were excluded from the ROI and whole brain analyses [*M*(SD) = 2.3 (5.13), min = 0, max = 22]. Because we tested right-handed subjects and language processing is mostly left lateralized in right-handed subjects, we only made ROIs in the left hemisphere.

A structural ROI analysis based on the cytoarchitectonical regions BA 4 (Geyer et al., 1996) and BA 6 (Eickhoff et al., 2005) was conducted to investigate the involvement of these entire structural regions in action language processing. Matlab based scripts for the Marsbar Toolbox (Brett et al., 2002) were used to extract the contrast values for verbs in non-literal and literal sentences. The data were further analyzed with an RMA using SPSS (Version 16.0 for Windows; SPSSInc. Chicago, IL, USA). A 2 × 2 RMA was calculated with the within subject factors BA (BA 4 and BA 6) and sentence type (literal, and non-literal).

For the functional ROI analysis we created a subject-specific 6 mm spherical ROI around the peak activated voxel in the left BA 6 (Eickhoff et al., 2005) and in the left BA 4 (Geyer et al., 1996), in response to “right hand” > “left hand” actions and “right foot” > “left foot” actions of the localizer task (thresholded at *p* < 0.001, uncorrected), using Matlab based scripts for the Marsbar Toolbox (Brett et al., 2002). To ensure that the ROIs did not overlap, the parts that fell into the other BA were cut off. This resulted in four unequal sized ROIs, therefore analyses on sentence type were conducted for each of the four ROIs. We extracted contrast values for the non-literal and literal hand and foot verbs and the last non-word of the baseline sentences.

To investigate whether action verb activation was organized in a somatotopic fashion, we conducted a 2 × 2 RMA for the ROIs (“foot area BA4”, “hand area BA4”, “foot area BA6”, “hand area BA6”) with the within subject factors sentence type (literal and non-literal) and extremity (hand sentence and foot sentence).

#### Additional whole brain analysis verbs and ROI analyses

A reviewer noticed that some probe words were action-related. Because of the relatively long BOLD response, brain activation to these probes may have been inseparable from the activation on the verb. Therefore, we conducted an additional whole brain analysis of the verbs and an additional ROI analysis. Eight verbs (two “non-literal foot”, four “literal foot”, one “non-literal hand”, and one “literal hand”) were excluded from the analyses. The results of this analysis can be found in the Section “Additional Whole Brain Analysis Verbs” and “Additional ROI Analyses” in the Appendix.

## Results

### Behavioral results

Overall accuracy was 82% (SD = 9%). The RMA of accuracy performance showed a main effect for condition [*F*(1,19) = 8.06, *p* < 0.01, $\eta_{p}^{2}=0.30$]. *Post hoc* within subjects difference contrasts revealed that participants scored higher on “non-literal hand” (*M* = 91%, SD = 7%) probe words than on “non-literal foot”, “literal foot”, “literal hand”, and “baseline” probe words [*M* = 80%, SD = 9%; *F*(1,19) = 40.14, *p* < 0.01, $\eta_{p}^{2}=0.68$]. The other comparisons of this difference contrast did not show significant differences (*p*s > 0.1).

The RMA of reaction time did not differ for the conditions “non-literal foot”, “non-literal hand”, “literal foot”, “literal hand”, and “baseline” (*p* > 0.1; *M* = 1216 ms, SD = 93 ms). Paired *t*-test for reaction time and accuracy revealed no indication of fatigue (*p*s > 0.05).

#### Non-literal sentence comprehension questionnaire

The average interrater reliability for the raters was found to be κ = 0.41 (SD = 0.31), suggesting a moderate agreement. Participants gave a correct meaning for 93% of the 100 non-literal expressions (SD = 0.07). The errors made by the participants, however, did not indicate that the non-literal sentences were comprehended as literal. Therefore we did not make a distinction between the incorrectly and correctly answered expressions in the analyses.

### Localizer task

The contrast “right hand movement” > “left hand movement” was associated with activation in the left postcentral gyrus (BA 4, *xyz* coordinates −36 −30 64). The contrast “right foot movement” > “left foot movement” was associated with activation in the left paracentral lobule (BA 4, *xyz* coordinates −6 −38 64). In the group analysis these contrasts showed clear somatotopy and the activation for hand and foot movement did not overlap.

For the functional ROI analysis we determined the peak voxel within BA 4 and BA 6 for each contrast. Right hand movement compared to left hand movement elicited activity in the left precentral gyrus (PreG), BA 6. This area was also more active for right foot over left foot activation, but spatially distinct (see Table 3). Right hand movement over left hand movement furthermore activated the left postcentral gyrus (PG, BA 4) and right foot movement over left foot movement activated the paracentral lobule (BA 4). These areas are associated with, respectively, the hand and the foot primary motor area (e.g., Hauk et al., 2004).

**Table 3:** **Mean peak coordinates localizer task**.

ROI	Peak average			Peak range			Region
	*x*	*y*	*z*	*x*	*y*	*z*	
Rfoot > Lfoot_BA6_	−9.67	−22	68.44	−44 to −2	−36 to 10	56 to 78	Precentral gyrus
Rhand > Lhand_BA6_	−39.33	−24	63.78	−44 to −28	−28 to −18	56 to 70	Precentral gyrus
Rfoot > Lfoot_BA4_	−7.33	−38.44	66.44	−14 to −4	−46 to −28	60 to 74	Paracentral lobule
Rhand > Lhand_BA4_	−38.44	−27.11	58.44	−42 to −34	−32 to −20	50 to 66	Postcentral gyrus

*Note: The highest average (*n* = 17) peak and range within Brodmann area 4 and 6 for right foot (Rfoot) versus left foot (Lfoot) and right hand (Rhand) versus left hand (Lhand) activation are shown and were thresholded at *p* < 0.001 uncorrected*.

### Whole brain analysis sentences

**Figure 1:**
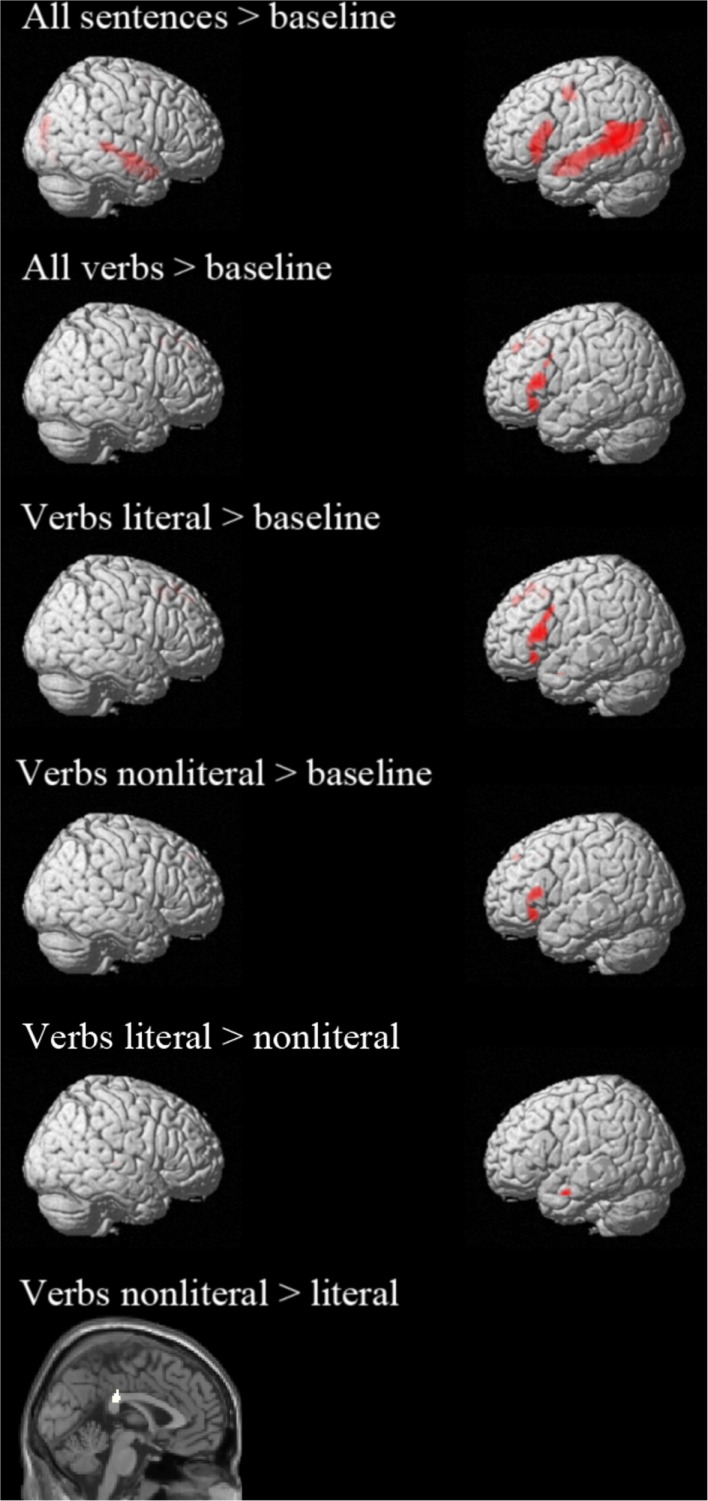
**Activation whole brain analyses**. All contrasts are thresholded at *p* < 0.05 FWE corrected.

The whole brain results are reported in Figure 1 and Table 4. Comparison of all sentences to the baseline (non-word) sentences revealed left lateralized activation in core language areas, namely the middle temporal gyrus (MTG) and inferior frontal gyrus (IFG). Furthermore, activity in the superior temporal gyrus (STG) and PreG was observed. These results show that the task successfully tapped into the language processing system (Xu et al., 2005).

**Table 4:** **Whole brain analysis**.

Contrast	Region	Approx BA	Extent	*Z*max	*x*	*y*	*z*
**SENTENCES**							
All sentences > baseline	L middle temporal gyrus	22	3606	>10	−52	−38	2
	R middle temporal gyrus	21	917	7.33	58	−8	12
	R cuneus	18	815	7.12	14	−88	26
	L inferior frontal gyrus	45	817	>10	−52	26	4
	L posterior cingulate	23	215	5.56	−8	−56	8
	L precentral gyrus	6	174	6.76	−48	−2	52
	L superior frontal gyrus	6	34	4.69	−6	6	62
**VERBS**							
All verbs > baseline	L inferior frontal gyrus	45	372	6.02	−48	26	10
	L inferior frontal gyrus			5.53	−48	28	−10
	L medial frontal gyrus	8	66	5.60	−4	18	48
	L superior frontal gyrus	8	73	5.18	−12	44	42
	L superior frontal gyrus			4.67	−10	30	50
	L inferior frontal gyrus	9	36	4.80	−50	16	28
Literal > baseline	L inferior frontal gyrus	45	531		−48	24	10
	L medial frontal gyrus (pre-SMA)	8	249	6.16	−4	18	48
	L superior frontal gyrus			5.14	−10	30	54
	L medial frontal gyrus			5.06	−10	44	42
	L superior frontal gyrus			4.47	−10	20	56
	L middle temporal gyrus	21	8		−48	4	−24
Non-literal > baseline	L inferior frontal gyrus	45	213	5.62	−48	26	10
	L inferior frontal gyrus			5.41	−50	28	−10
	L superior frontal gyrus		19	4.76	−12	44	44
	L cingulate gyrus	32	9	4.69	−6	20	46
Literal > non-literal	L middle temporal gyrus	21	21	5.11	−48	0	−24
	R thalamus		11	4.67	18	−24	4
Non-literal > literal	Cingulate gyrus	23	24	4.63	0	−30	26

*Note: All contrasts are thresholded at *p* < 0.05 FWE corrected*.

### Whole brain analysis verbs

To check whether whole brain analysis of the verbs also showed activation of the language system, we looked at the contrast “all verbs” > “baseline”. This contrast shows activation in left IFG, left superior frontal gyrus (SFG), and left medial frontal gyrus [MFG, pre-supplementary motor area (pre-SMA)] (see Figure 1; Table 4). From these results it can be concluded that the verb analysis successfully tapped into the language processing system (Xu et al., 2005).

Action words in literal sentences and in non-literal sentences were compared to the baseline condition to investigate whether the motor activation is verb-based or dependent upon the context of a sentence. The contrast “literal” > “baseline” was associated with activation in left IFG, left MFG (pre-SMA), left SFG, left MTG. Activation of the pre-SMA suggests that motor regions were recruited more when reading literal sentences compared to baseline sentences. The contrast “non-literal” > “baseline” was associated with activation in the left IFG and left SFG and left cingulate gyrus (CG). Similar CG activation has been found in other studies (Tettamanti et al., 2005; Desai et al., 2010). The CG is thought to have a role in providing the emotional connotation of colorful figurative language (Proverbio et al., 2009). This contrast activated only language regions and no motor regions.

To contrast the literal and non-literal conditions the same concrete verbs, which were used in both conditions, were compared. Therefore, the contrast only reflected differences in non-literal and literal sentence context. The comparison “literal” > “non-literal” showed activation in the left MTG and the right thalamus. The reverse contrast (“non-literal” > “literal”) showed activation in the CG. The contrasts “literal foot” > “non-literal foot”, “literal hand” > “non-literal hand”, “hand” > “foot”, and the reversed contrasts did not activate any regions at *p* < 0.05 with FWE correction. Only compared to baseline sentences did the literal sentences show pre-SMA activation. This analysis was not sensitive enough to detect differences in motor activation in the contrast “literal” > “non-literal”. See Section “Additional Whole Brain Analysis Verbs with a Less Conservative Threshold” in the Appendix for an additional analysis of these contrasts with a less conservative threshold.

### ROI analyses

**Figure 2:**
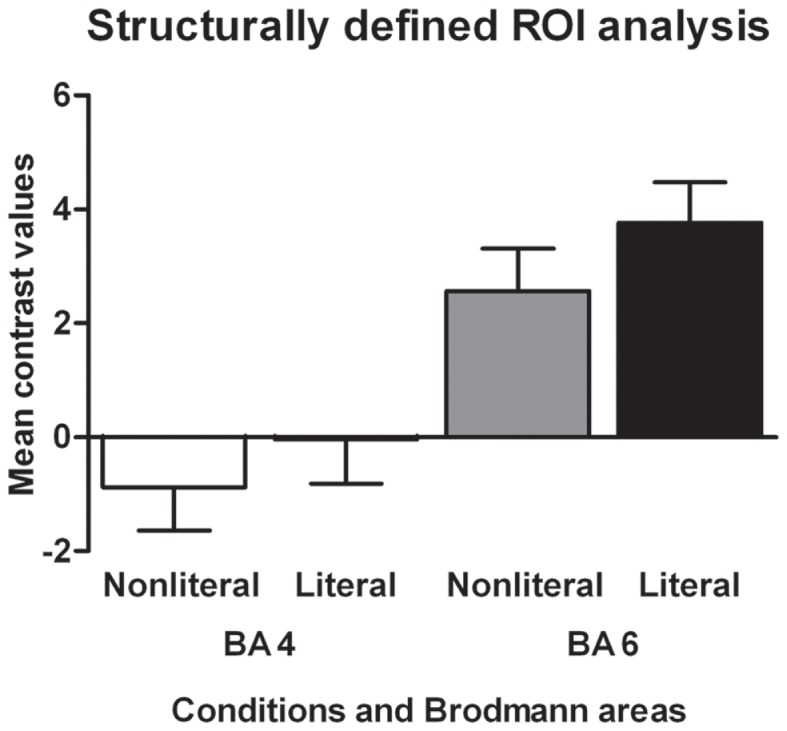
**Structurally defined ROI analysis**. The mean contrast values compared to an implicit baseline are shown for each Brodmann area and sentence type.

The results of the structurally defined ROI analysis are shown in Figure 2. The RMA based on the BAs showed a main effect of sentence type [*F*(1,17) = 7.01, *p* < 0.05, $\eta_{p}^{2}=0.29$]. Literal sentences were associated with more activation in both BA 4 and BA 6 than non-literal sentences. BA also showed a main effect, BA 6 was associated with more activation in response to the verbs than BA 4 [*F*(1,17) = 51.17, *p* < 0.001, $\eta_{p}^{2}=0.75$] and the interaction between BA and sentence type was not significant (*p* = 0.18).

**Figure 3:**
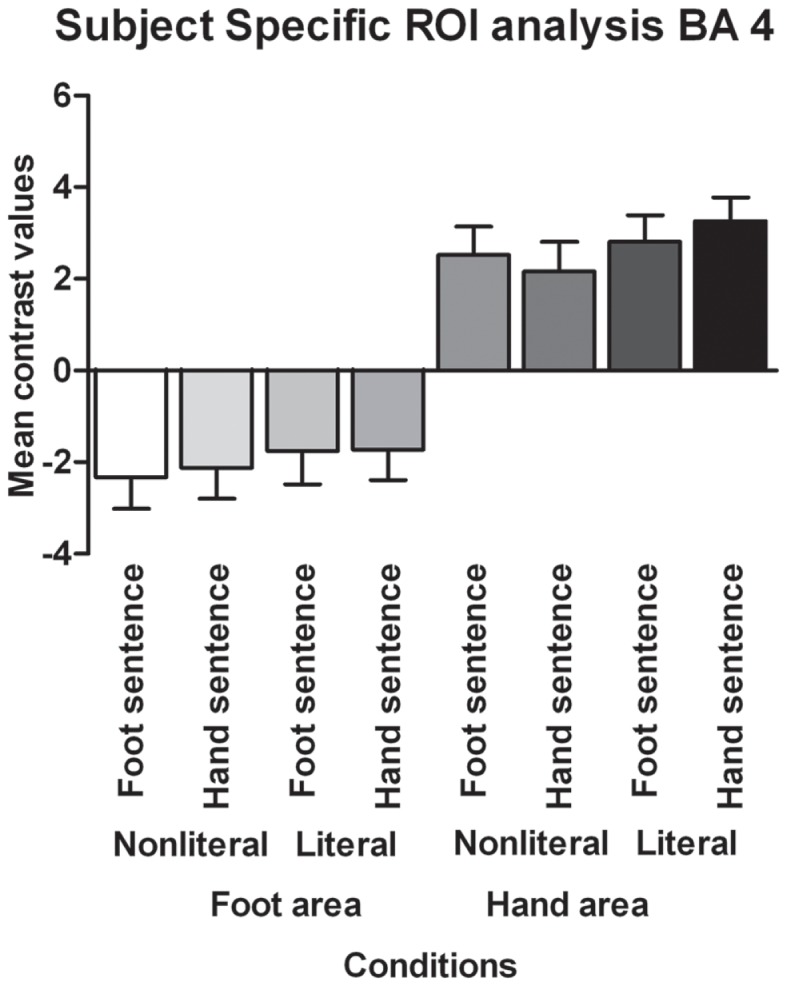
**Functionally defined, subject-specific ROI analysis for the foot area and hand area in BA 4**. The mean contrast values compared to an implicit baseline are shown for each sentence type and extremity.

In order to test for semantic somatotopy we conducted a subject-specific functional ROI analysis based on the motor area localizer task combined with the structurally defined ROIs BA 4 and BA 6. Contrary to the somatotopy prediction we did not find a main effect of extremity (*p* = 0.75), nor an interaction between sentence type and extremity for the BA 4 foot area (*p* = 0.80). In addition, we found no main effect of sentence type (*p* = 0.24, see Figure 3).

Contrary to the somatotopy prediction we did not find a main effect of extremity (*p* = 0.88), nor an interaction between sentence type and extremity for the BA 4 hand area (*p* = 0.23). Furthermore, a main effect of sentence type was found [*F*(1,17) = 4.82, *p* < 0.05, $\eta_{p}^{2}=0.22$]. Verbs in literal sentences engaged the BA 4 hand area more than verbs in non-literal sentences.

**Figure 4:**
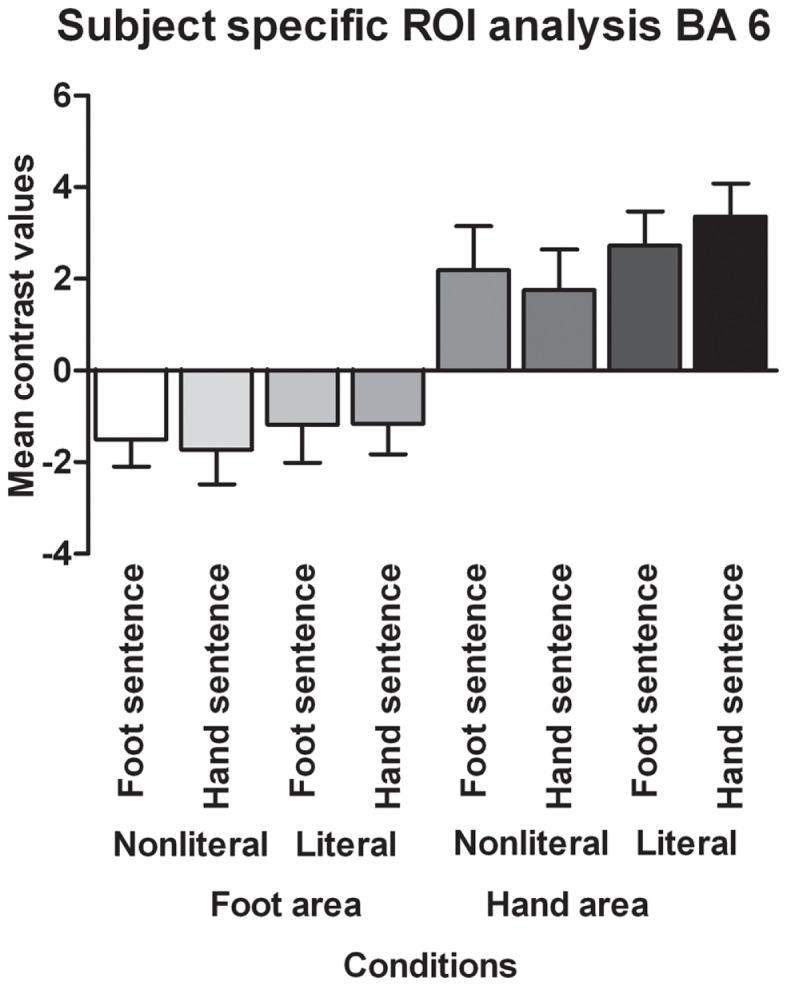
**Functionally defined, subject-specific ROI analysis for the foot area and hand area in BA 6**. The mean contrast values compared to an implicit baseline are shown for each sentence type and extremity.

Contrary to the somatotopy prediction we did not find a main effect of extremity (*p* = 0.82, see Figure 4), nor an interaction between sentence type and extremity for the BA 6 foot area (*p* = 0.69). In addition, no main effect of sentence type was found (*p* = 0.10). For the BA 6 hand area we found no main effect of extremity (*p* = 0.82), nor an interaction between sentence type and extremity (*p* = 0.10). A significant main effect was found for sentence type [*F*(1,17) = 6.30, *p* < 0.05, $\eta_{p}^{2}=0.27$]. Verbs in literal sentences engaged the BA 6 hand area more than verbs in non-literal sentences.

## Discussion

We investigated the involvement of the motor cortex in comprehending action verbs in non-literal and literal sentences. The whole context was presented before the verb so that it was clear to the comprehender whether a particular sentence was literal or non-literal before the verb was read. As predicted by the weak embodiment hypothesis, we found that the amount of motor activation depended on the context of a sentence. This does not signify that verbs in non-literal sentences are not comprehended as well as verbs in literal sentences. It is likely that these verbs are processed (partly) by a semantic system. These results do not support the strong view of embodied cognition, according to which the motor cortex should be activated regardless of sentential context. We were not able to test whether action verbs in non-literal sentences that were not understood properly engaged the motor system more than action verbs in properly comprehended non-literal sentences, because the error rate was too low.

Our study, including the functionally and structurally defined ROIs and verbs as the last word of the sentence, shows results that are in line with previous studies (Aziz-Zadeh et al., 2006; Raposo et al., 2009; Desai et al., 2010; and for literal language: Tettamanti et al., 2005). In other words, we also find that action verbs embedded in literal sentences engage the PM to a larger extent than do action verbs in non-literal sentences. However, our results do not support the somatotopy hypothesis for action words, unlike Hauk et al. (2004); Tettamanti et al. (2005); Aziz-Zadeh et al. (2006); Boulenger et al. (2009); and Raposo et al. (2009).

The contrast “literal” > “baseline” was associated with activation in the pre-SMA. The contrast “literal” > “non-literal” with a less conservative threshold also was associated with activation in the pre-SMA (see Section “Additional Whole Brain Analysis Verbs with a Less Conservative Threshold” in the Appendix). The finding of pre-SMA activation in response to action words is in line with the findings by Postle et al. (2008). They used single action verbs (e.g., *kick*), which could have been perceived as instructions (e.g., *kick!*) that enabled the retrieval of relevant motor programs. The retrieval of information required for motor planning was associated with activation in the pre-SMA. Likewise, in our study the pre-SMA activation could signify a role in maintaining abstract representations of action verbs. However, in our case the pre-enactment of future experiences was not related to instructional cues, but action verbs embedded in literal sentences activated the pre-SMA for partial preparation of the described action. Because of its link to the ventral premotor areas, it is considered to have a more cognitive role in the formation and retrieval of motor sequences, next to a role in motor functions (Picard and Strick, 2001). Rueschemeyer et al. (2010) also found activation in the pre-SMA for functionally manipulable words (e.g., pen, cup), but not for volumetrically manipulable words (e.g., bookend, clock). Words like *pen* are thought to have stronger associations to a specific type of motor information than words like *clock*. This link between (action) word processing and a general motor association may be supported by pre-SMA.

The structurally defined ROI analyses did not confirm that verbs in non-literal and literal sentences differentially engage BA 4 (M1) or 6 (PM), however in general BA 6 was more active during language processing than BA 4. Action verbs in literal or non-literal sentences were not found to be associated with a somatotopic organization. As mentioned in the introduction, there are several concerns with the conclusion that semantic processing of action verbs related to different body parts evokes somatotopically specific activity in motor regions. First, most studies find only partial overlap between the read and performed hand/foot related actions (see Postle et al., 2008). Second, some studies do not report a somatotopic organization for action words. For example, Postle et al. (2008) found no somatotopically organized activation for action words. Nor did they find overlap between activation for action words and execution or observation of the congruent effectors. How can these contradictory findings be explained? A reason that somatotopic variations in activation in the PM are found may be that M1 has a clearer somatotopic organization than the PM (Fernandino and Iacoboni, 2010). The degree of somatotopic organization thus depends on the extent to which the PM is activated by actions performed with different effectors. Because action simulation activates the PM (and not M1) and this area has a more opaque somatotopic organization, a somatotopic organization is likely more difficult to find. Furthermore, tasks and contrasts used in studies may also explain some of the variability in PM activation, because each task and contrast is likely to vary in the degree of PM involvement.

Another reason for the inconsistencies in semantic somatotopy may be that the organization of actions is more goal-related than effector-related. In one study participants had to trace zigzag patterns with their big toe or index finger. Toe movement activated the supplementary motor area (SMA) and index finger movement activated dorsolateral PM (Rijntjes et al., 1999). However, when subjects traced their signatures with either the index finger or big toe, effector-independent activation in the dorsolateral PreG was found. Making a signature with the toe recruited the same regions that usually control signing with the hand. These results show that, in addition to an effector-specific organization, a goal-related organization exists in the brain. These findings may be an explanation for the lack of evidence for a semantic somatotopic organization of the motor cortex we found, because action sentences may be considered goal-oriented, independent of body part, rather than effector-oriented. In the current study, the stimuli were not designed to investigate this. We compared hand sentences with foot sentences. The hands are used to move and manipulate objects (for example to the mouth), whereas the feet are typically used for locomotion or to propel objects away from the body. Future research could focus on goal specificity.

The current study shows that PM is involved in action language processing, but the debate whether PM is necessary for action language comprehension is not over yet. Mahon and Caramazza (2008), suggest a theory of secondary embodiment, the grounding by interaction hypothesis. According to this theory motor activation is epiphenomenal; it may play a supportive but not a necessary role in representing concepts. A limitation of the current study is that we did not vary the presentation time between the verb and the probe. Therefore the activation related to the verb may have been inseparable from the activation to the probe. We attempted to solve this problem by taking the verbs out of the analysis that had an action-related probe (see Additional Whole Brain Analysis Verbs and Additional ROI Analyses in Appendix). Moreover, we calculated the variance inflation factor (VIF) with Matlab based scripts (http://wagerlab.colorado.edu/tools). And this showed an VIF of 8, which is considered acceptable (Myers, 1990).

In conclusion, motor activation is context dependent. When we read a sentence in which the motor properties of a word are of importance, motor areas are recruited. When the motor properties are not important, such as in non-literal sentences, the motor areas are less activated. Our results support the weak view of embodied cognition.

## Conflict of Interest Statement

The authors declare that the research was conducted in the absence of any commercial or financial relationships that could be construed as a potential conflict of interest.

## Figures and Tables

**Figure A1:**
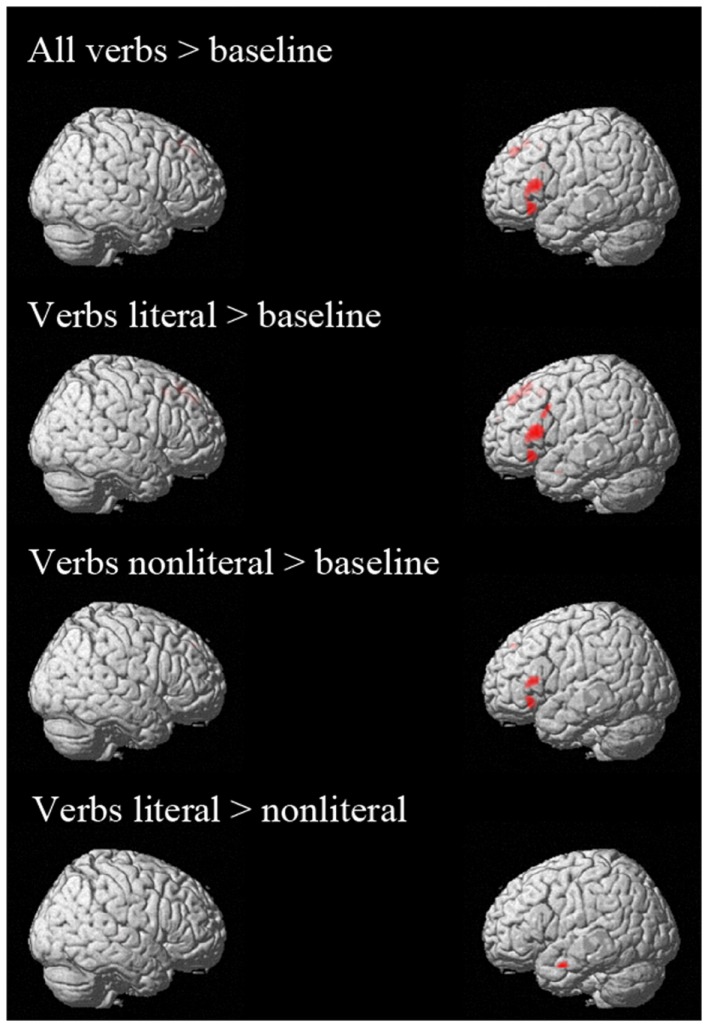
**Activation additional whole brain analyses**. All contrasts are thresholded at *p* < 0.05 FWE corrected.

**Figure A2:**
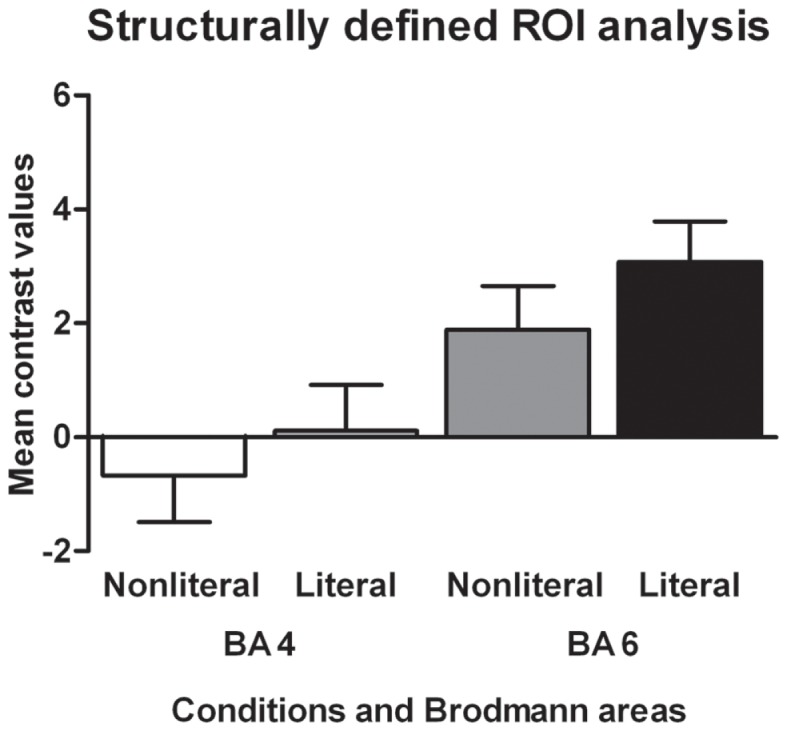
**Additional structurally defined ROI analysis**. The mean contrast values compared to an implicit baseline are shown for each Brodmann area and sentence type.

**Figure A3:**
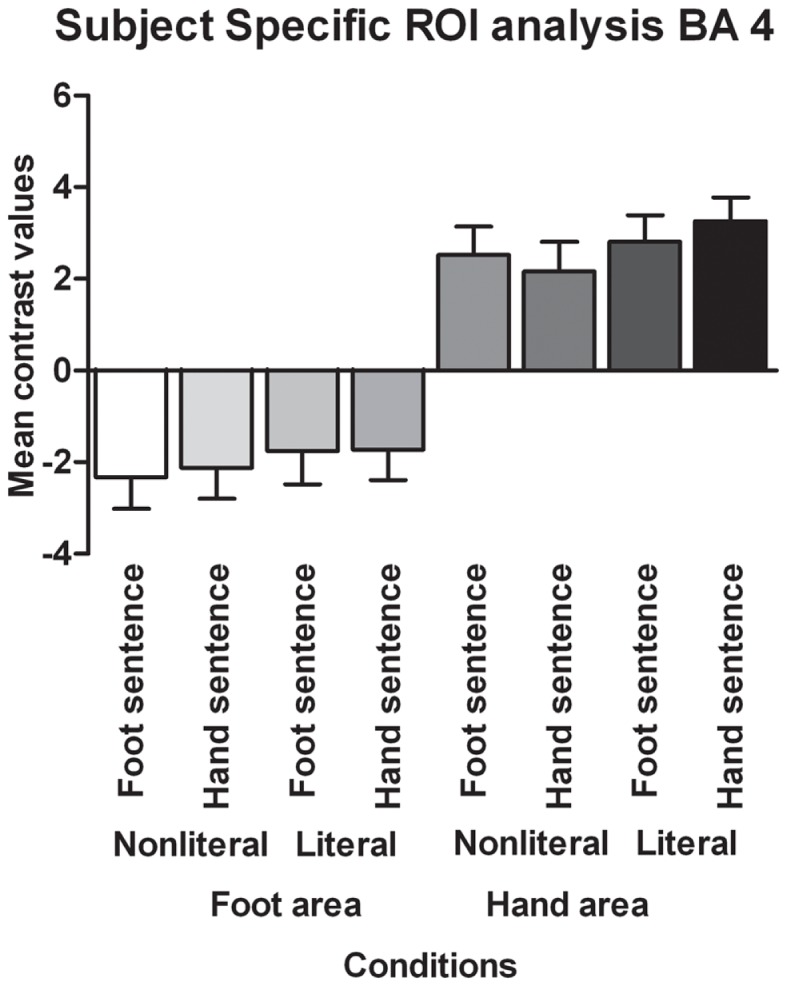
**Additional functionally defined, subject-specific ROI analysis for the foot area and hand area in BA 4**. The mean contrast values compared to an implicit baseline are shown for each sentence type and extremity.

**Figure A4:**
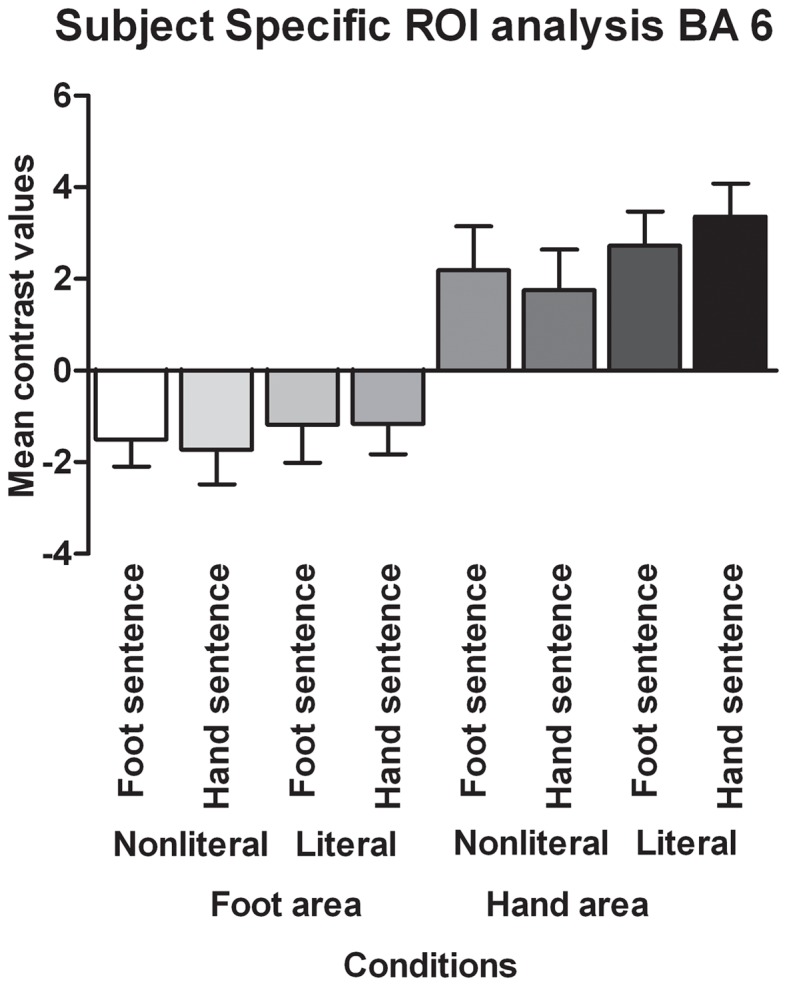
**Additional functionally defined, subject-specific ROI analysis for the foot area and hand area in BA 6**. The mean contrast values compared to an implicit baseline are shown for each sentence type and extremity.

**Figure A5:**
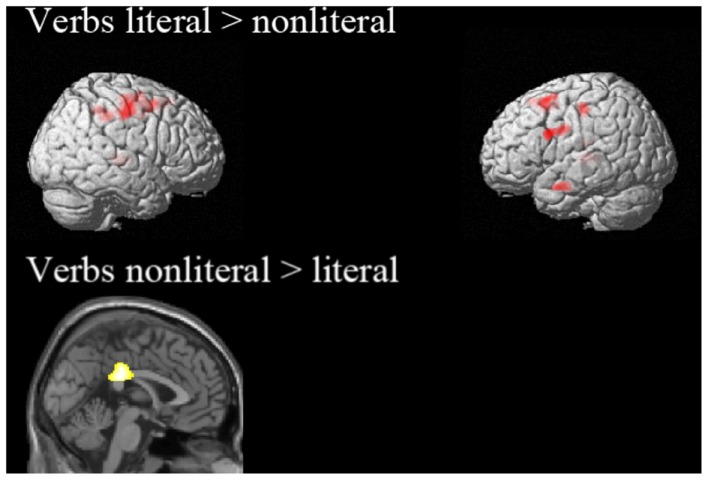
**Activation additional whole brain analyses with a less conservative threshold**. All contrasts are thresholded at *p* < 0.001 uncorrected, cluster extent 100 voxels.

## Appendix

We conducted an additional whole brain analysis of the verbs and an additional ROI analysis, where eight verbs (two “non-literal foot”, four “literal foot”, one “non-literal hand”, and one “literal hand”) were excluded from the analyses, because these probe words were action-related (see Section “Additional whole brain analysis verbs” and “Additional ROI analyses”). In the last section of the Appendix an additional analysis is described with a less conservative threshold, including the eight verbs.

### Additional whole brain analysis verbs

The contrast “all verbs” > “baseline” was associated with activation in left IFG, left SFG, and left MFG (see Figure A1; Table A1). From these results it can be concluded that the verb analysis successfully tapped into the language system (Xu et al., 2005).

**Table A1:** **Additional whole brain analysis**.

Contrast	Region	Approx BA	Extent	*Z*max	*x*	*y*	*z*
**VERBS**							
All verbs > baseline	L inferior frontal gyrus	45	280	5.80	−48	26	10
	L superior frontal gyrus	8	98	5.18	−12	44	42
	L medial frontal gyrus	8	28	5.10	−4	18	48
Literal > baseline	L inferior frontal gyrus	45	265	5.86	−48	24	10
	L superior frontal gyrus		165	5.31	−8	32	52
	L medial frontal gyrus (pre-SMA)	8	82	5.76	−4	18	48
	L inferior frontal gyrus	47	69	5.53	−48	26	10
	L middle frontal gyrus	9	57	4.90	−50	14	30
	L middle temporal gyrus	21	6	4.63	−48	4	−24
Non-literal > baseline	L inferior frontal gyrus	45	91	5.39			
	L inferior frontal gyrus	47	48	5.22			
	L superior frontal gyrus		13	4.77			
Literal > non-literal	L middle temporal gyrus	21	23	5.23	−48	0	−24

*Note: All contrasts are thresholded at *p* < 0.05 FWE corrected*.

The contrast “literal” > “baseline” was associated with activation in left IFG, left MFG (pre-SMA), left SFG, and left middle frontal gyrus (MidFG). The activation related to these two contrasts was similar to the activation found in the previous whole brain analysis. Activation of the pre-SMA suggests that motor regions were recruited more when reading literal sentences compared to baseline sentences. The contrast “non-literal” > “baseline” was associated with activation in the left IFG and left SFG. In the previous whole brain analysis we found similar activation and in addition CG activation. This contrast activated only language regions and no motor regions.

The comparison “literal” > “non-literal” showed activation in the left MTG. In the previous whole brain analysis we found similar activation for this contrast plus right thalamus activation. The reverse contrast (“non-literal” > “literal”) did not activate any regions at *p* < 0.05 with FWE correction. In the previous whole brain analysis this contrast was associated with CG activation.

The contrasts “literal foot” > “non-literal foot”, “literal hand” > “non-literal hand”, “hand” > “foot”, and the reversed contrasts did not activate any regions at *p* < 0.05 with FWE correction. This finding is similar to the previous whole brain analysis.

### Additional ROI analyses

The results of the structurally defined ROI analysis are shown in Figure A2. The RMA based on the structurally defined ROIs showed a main effect of sentence type [*F*(1,17) = 6.48, *p* < 0.05, $\eta_{p}^{2}=0.28$]. Literal sentences were associated with more activation in both BA 4 and BA 6 than non-literal sentences. BA also showed a main effect, BA 6 was associated with more activation in response to the verbs than BA 4 [*F*(1,17) = 42.77, *p* < 0.001, $\eta_{p}^{2}=0.75$] and the interaction between BA and sentence type was not significant (*p* = 0.16). These findings are similar to the previous ROI analysis.

The subject-specific functional ROI analysis based on the motor area localizer task combined with the structurally defined ROIs BA 4 and BA 6 did not show a main effect of extremity (*p* = 0.90) or an interaction between sentence type and extremity for the BA 4 foot area (*p* = 0.88). In addition, we found no main effect of sentence type (*p* = 0.30, see Figure A3).

Contrary to the somatotopy prediction we did not a main effect of extremity (*p* = 0.74), nor an interaction between sentence type and extremity for the BA 4 hand area (*p* = 0.12). In addition, we found a near-significant main effect of sentence type (*p* = 0.06). These findings are similar to the previous ROI analysis. The main effect of sentence type in BA 6 shows a marginally significant effect in this analysis and a significant effect in the previous ROI analysis.

For the BA 6 foot area we found no significant main effect for extremity (*p* = 0.72), nor a significant interaction between sentence type and extremity (*p* = 0.35; see Figure A4). In addition, we found no main effect of sentence type (*p* = 0.11). This means that within the BA 6 foot area there also is no evidence of semantic somatotopy.

For the BA 6 hand area we found a near-significant interaction between sentence type and extremity (*p* = 0.06). No significant main effect was found for extremity (*p* = 0.76). The main effect of sentence type was significant [*F*(1,17) = 6.05, *p* < 0.05, $\eta_{p}^{2}=0.26$]. The results of this analysis is similar to the previous ROI analysis.

Contrary to the somatotopy prediction, no significant main effect of extremity or an interaction between sentence type and extremity was found. In other words, hand action verbs did not activate the hand area more than did foot action verbs. Nor did foot action verbs activate the foot area more than did hand action verbs. Of primary interest was the motor activation in response to action verbs in literal versus non-literal sentences. Only in the BA 6 hand area the literal sentences were associated with more motor activation than the non-literal sentences.

### Additional whole brain analysis verbs with a less conservative threshold

Based on the comparison “literal” > “baseline” and the ROI analysis, we expected to find motor activation for the contrast “literal” > “non-literal”, therefore we looked at this contrast with a less conservative threshold of *p* < 0.001 uncorrected with a cluster extent of 100 voxels (See Figure A5; Table A2). This contrast was associated with activation in left MTG, left MFG (pre-SMA), bilateral thalamus, right PreG, and left IFG. Next to activation of language processing areas, we find activation in the left pre-SMA, consistent with the ROI analyses and the contrast “literal” > “baseline”.

**Table A2:** **Additional whole brain analysis, with a less conservative threshold**.

Contrast	Region	Approx BA	Extent	*Z*max	*x*	*y*	*z*
**VERBS**							
Literal > non-literal	R Thalamus		312	4.67	18	−24	4
	L Medial frontal gyrus (pre-SMA)	6	339	4.41	−4	14	50
	R Precentral gyrus		1020	4.29	32	−18	46
	L Thalamus		383	4.11	−16	−24	4
	L Inferior frontal gyrus		231	3.83	−50	10	26
Non-literal > literal	Cingulate gyrus	23	341	4.63	0	−30	26

*Note: All contrasts are thresholded at *p* < 0.001 uncorrected, extent threshold of 100 voxels*.

The reverse contrast showed activation in CG. The CG is thought to have a role in providing the emotional connotation of colorful figurative language (Proverbio et al., 2009). This contrast did not activate any motor regions.

The contrasts “literal foot” > “non-literal foot”, “literal hand” > “non-literal hand”, “hand” > “foot”, and the reversed contrasts did not activate any regions at *p* < 0.001 uncorrected.
